# Evaluation of health related quality of life in children with immune thrombocytopenia with the PedsQL™ 4.0 Generic Core Scales: a study on behalf of the pays de Loire pediatric hematology network

**DOI:** 10.1186/1477-7525-11-193

**Published:** 2013-11-13

**Authors:** Marion Strullu, Josué Rakotonjanahary, Eliane Tarral, Christophe Savagner, Caroline Thomas, Françoise Méchinaud, Yves Reguerre, Sylvaine Poignant, Arnaud Boutet, Joachim Bassil, Dominique Médinger, Emmanuel Quemener, Nancy L Young, Petronela Rachieru, Robert J Klaassen, Isabelle Pellier

**Affiliations:** 1 Service d’Immuno-Hémato-Oncologie Pédiatrique, Hôpital Mère-Enfant, CHU de Nantes, Nantes, France, UE; 2 Service d’Immuno-Hémato-Oncologie Pédiatrique, CHU d’Angers, 4 rue Larrey, 49933 Angers, Cedex 09, France, UE; 3 Service de Pédiatrie, CH Le Mans, Le Mans, France, UE; 4 Service de Néonatalogie, CHU d’Angers, Angers, France, UE; 5 Service de Pédiatrie, CH Cholet, Cholet, France, UE; 6 Service de Pédiatrie, CH Saint-Nazaire, Saint-Nazaire, France, UE; 7 Service de Pédiatrie, CH Laval, Laval, France, UE; 8 Service de Pédiatrie, CH La Roche sur Yon, La Roche sur Yon, France, UE; 9 Centre de recherche clinique d’Angers, Angers, France, UE; 10 School of Rural and Northern Health, Laurentian University, Sudbury, Canada; 11 Department of Pediatrics, Children's Hospital of Eastern Ontario, Ottawa, Canada; 12 Inserm, Unité 892, Centre de Recherche en Cancérologie Nantes-Angers, Angers, France, UE; 13 Inserm CIE 5, Hôpital Robert Debré, Paris, France, UE

**Keywords:** Health related quality of life, Immune thrombocytopenia, Childhood, PedsQL™ 4.0 Generic Core Scales

## Abstract

**Background:**

Immune thrombocytopenia (ITP) is a childhood disorder that is often life-altering for children and their parents. Health related quality of life (HRQL) has never been chronologically monitored in children with ITP. We initiated a prospective study to assess HRQL from diagnosis to six months and define factors that influence this outcome in children with ITP**.**

**Methods:**

73 children with acute ITP aged from 2 to 18 years were prospectively enrolled in the study. According to the presence of bleeding, they were or were not given a 4-day course of corticosteroid treatment. The PedsQL™ 4.0 Generic Core Scale was completed by children and parents upon their inclusion in the study and 6 months after diagnosis.

**Results:**

Over the six month period, quality of life improved in terms of their global, physical and psychosocial well-being for 54.5%, 35.6% and 36.2% of patients respectively. This improvement is clinically relevant compared to scores at diagnosis, corresponding at least to a minimal clinically important difference (MCID). Factors such as sex, age, platelet count, bleeding scores, bone marrow aspiration and persistence of ITP at 6 months were not significantly associated with HRQL scores. However, preceding viral infection was identified to have an impact on HRQL.

**Conclusions:**

This first longitudinal study assessing HRQL in children with ITP reveals a global improvement in PedSQL™ 4.0. However, these results should be considered with caution since our data also confirm that self-report HRQL scores are not influenced by any analyzed biologic or clinical parameters. Others tools, such as Kids’ ITP Tools, would probably be required to assess the HRQL of this population.

**Trial registration:**

Trial registration clinical trials.gov Identifier: NCT00331357.

## Background

Immune thrombocytopenia (ITP) is a relatively uncommon auto-immune disorder in childhood (estimated incidence of 4 to 5 per 100,000 person-years) characterized by an acquired decrease in circulating platelets [1,2]. Severe morbidity and mortality are rare and occur in approximately 0.1% to 3% of patients [3,4]. In more than 70% of cases, the disease resolves itself within six months, regardless of the treatment given [5]. The decision to treat and the type of treatment are controversial, since among the different medications available, none has proven its superiority regarding reduction in bleeding complications or transition to chronicity [6-9]. Furthermore, profound thrombocytopenia, the risk of intracranial bleeding, and the appearance of bruises may seriously influence lifestyle, school functioning and physical activities and frequently result in anxiety in children and parents. Therefore, since the benefit-risk ratio of current medications is unclear [7,8] and as the psychological and the physical burden of the disease is highly variable, it appears necessary to develop new tools to better understand the exact impact of the disease on daily life. Although treatment decision-making depends essentially on the physician’s experience, it seems of interest to include in this reflection a measurement of quality of life. In this context, evaluation of health related quality of life (HRQL) could be considered in ITP, as in numerous chronic diseases, as a method to provide information about the effects of medical interventions [10,11].

Recently, an International Working Group of recognized experts defined standard terminology of primary ITP and criteria of outcome and response [12]. Special attention was paid to the importance of developing specific HRQL tools to assess the impact of ITP on the child’s life. To date, few studies assessing this issue in ITP are available [13,14]. A disease-specific measure of HRQL for children with ITP, the Kids’ ITP Tools (KIT), was validated first in North America [15]. This measure has recently received cross-cultural validation in several countries and shows promise for use in international trials [16]. Contrary to disease-specific tools, generic measures do not focus on the specific troubles associated with the disease, but are applicable across different populations and health states, allowing comparison with the healthy population. Both are complementary and thus recommended for the full assessment of HRQL [17]. While processing the validation of the KIT, we initiated a study on a different pediatric cohort which is the first prospective attempt to assess the well-being of children with ITP from diagnosis to six months with the generic PedsQL™ 4.0 Generic Core Scales. The following key questions were addressed: is the HRQL of patients with ITP impaired at diagnosis and six months later, in comparison with the general population? What are the factors that impact on evolution of HRQL in patients with ITP?

## Methods

### Patients

From January 2007 to May 2009, 73 children and adolescents aged from 2 years to 18 years with ITP were enrolled in this prospective study. They were recruited in seven pediatric centers of the region Pays de Loire, France. Patients were eligible if parental consent was obtained and if they did not have any concomitant disease that could impact their quality of life. If they were old enough, the child’s assent was also obtained. The study was approved by the comité de protection des personnes.

A single reference protocol was set up within each hospital, using a short-term corticosteroid therapy (4 mg/kg per day during 4 days, with a maximum dose of 180 mg per day) as the standard first-line treatment. The severity of ITP was stratified into four distinct groups, according to the platelet count and the existence of hemorrhagic signs, based on the consensus developed by the Intercontinental Childhood ITP Study Group (ICIS) (Table 1) [18] which we chose over the Buchanan score although the latter remains widely used [19]. Stages I and II do not require treatment and were subjected to clinical observation. Patients with stage III could either receive a 4-day course of corticosteroid or be subjected to simple surveillance, as judged appropriate by the treating physician. For stage IV, the first-line treatment was methylprednisolone or prednisone. Bone marrow aspiration was not required before initiation of corticosteroid therapy if an experienced practitioner could attest to the absence of signs of malignancy with normal blood cell smear. In cases of failure to respond to the initial corticosteroid treatment, or secondary recurrence, an infusion of intravenous immunoglobulin (IVIG) at 0.8 to 1g/kg was administered at 48-hour intervals if necessary. After diagnosis (D1), patients had outpatient follow-up visits at 1 month (M1), 3 months (M3) and 6 months (M6). Complete blood count, physical examination, adverse effects and ICIS bleeding scores were collected at each visit. Complete response was defined as a platelet count above 100.10^9^/L. If the patient relapsed, observation, corticosteroid therapy or IVIG could be considered according to the ICIS score, previous treatment, and the physician’s practice.

**Table 1:** Intercontinental Childhood ITP Study group (ICIS) staging

**Stage/Bleeding**	**Description**
**I**	No bleeding.
	*And* Platelet count above 20 × 10^9^/L.
**II**	Bruising, petechiae of skin or occasional epistaxis.
	Little or no interference with daily living.
	*And* Platelet count above 10 × 10^9^/L.
**III**	Bruising, petechiae of skin.
	Mucosal lesions.
	Epistaxis requiring wicking and hemorrhage.
	*And* Platelet count above 10 × 10^9^/L.
**IV**	Bleeding: epistaxis, melena and/or hemorrhage requiring hospitalization and/or transfusion.
	Serious interference with quality of life.
	*Or* Platelet count below 10 × 10^9^/L and/or haemoglobin decrease over 2 g/dl.

### Quality of life

Quality of life was assessed by the PedsQL™ 4.0 Generic Core Scales. The PedsQL™ 4.0 Generic Core Scales is a generic questionnaire that measures HRQL by a self-report and/or a parent-proxy report for patients aged from 2 to 18 years: HRQL of patients under 5 years is exclusively measured by the parent proxy-report [20]. It was completed independently, at D1, M1, M3 and M6, by parents and children. D1 corresponds to the diagnosis day and also, generally, to the initiation of treatment. For younger children, an assistant was appointed to explain instructions and answer questions. Parent proxy-reports and age-specific child self-reports were similar versions, with formulations appropriate to the understanding of the responder. This questionnaire has been widely used in previous studies and its validity and reliability has been well demonstrated [20-22].

### Statistical analyses

We compared ITP children and parents mean PedsQL scores to previously published scores for the healthy reference population [20,21,23]. Independent sample t-tests were used to compare scores. Impaired HRQL was defined as a PedsQL score at least 1 standard deviation (SD) below the healthy sample mean score. Original norm data from a sample of healthy children was used as reference and scores from french healthy children were provided for information [21-24].

The percentages of scores at the extremes of the score scale, the floor value (minimum possible score i.e. 0) and the ceiling value (maximum possible score i.e. 100), were described to explore the ability for ITP children to distinguish variations in quality of life. The ceiling effect is defined as an inability for children who had the maximum possible score to get a better score when a subsequent assessment is performed (the floor effect is the same phenomenon but with scores at the low end).

Intraclass Correlation coefficients (ICC) were used to describe agreement between child self-report and parent proxy-report across age subgroups. Intraclass Correlation Coefficients are designated as ≤ 0.40 for poor to fair agreement, 0.41–0.60 for moderate agreement, 0.61–0.80 for good agreement, and 0.81–1.00 for excellent agreement.

The minimal clinically important difference (MCID) concept was used to explore improvement in quality of life between diagnosis and M6. MCID is defined as the smallest difference in a PedsQL score of a domain of interest that each patient perceives to be beneficial and that would mandate a change in the patient’s management [21]. MCID scores were computed by using the Standard Error of Measurement (SEM) of scores at diagnosis. SEM was computed by multiplying the standard deviation by the square root of 1-alpha (Cronbach alpha coefficient). Excellent agreements between the SEM and MCID were shown in the literature [25,26]. Improvement in quality of life was defined as the score at M6 corresponding to the score at diagnosis with an improvement at least equal to the value of the MCID score.

Potential predicting factors that impact improvement in quality of life at M6 as previously defined were determined by univariate logistic regression. The associations are presented as beta coefficients with standard error (SE) derived from logistic regression. Given the exploratory nature of this analysis, we did not perform multivariate analyses. Statistical analyses were conducted using Stata software (version 12.1; StataCorp, Texas).

## Results

### Characteristics of patients

Between January 2007 and May 2009, 97 children were diagnosed with primary ITP in the region Pays de Loire. Among them, 16 children under 2 years of age were not eligible to be enrolled in the study because of age. Ultimately, 81 families were asked to complete the proxy and/or self-report version of the PedsQL™ 4.0 Generic Core Scales.

For the 34 children < 5 years, HRQL was exclusively measured by the parent proxy-report. Among the 47 self-reports of children > 5 years and the 81 parent-proxy reports, 8 reports in each group were partially uncompleted for more than 50% of the items. Therefore, according to the PedsQL™ 4.0 recommendations, these reports were considered as ineligible for the study. Finally, 73 children were included for analysis in the study: 34 aged 2 to 5 years, 39 aged 5 to 18 years, and 73 parents. Major characteristics of the 73 children at diagnosis are summarized in Table 2.

**Table 2:** Characteristics of ITP patients at diagnosis

**Characteristics**	**Diagnosis**
**Patients, n**	73
**Median age, y (IQ)**	7.6 (2 – 17.7)
**Gender, n**	
Male	33 (45%)
Female	40 (55%)
**Preceding viral infection, n (%)**	44 (60.2%)
**ICIS scores, n (%)**	
Score I	5 (6.9%)
Score II	22 (30.1%)
Score III	2 (2.7%)
Score IV	44 (60.3%)
Initial place of treatment, n (%)**	
In patient	52 (71.3%)
Observation unit*	10 (13.7%)
Outpatient	11 (15%)
**Initial bone marrow examination, n (%)**	11 (15%)
**Initial treatment, n (%)**	
No treatment	25 (34.2%)
Corticosteroid	39 (53.5%)
IVIG	9 (12.3%)

n: number of patients; IVIG: intravenous immunoglobulin;

*An observation unit is a unit of short hospital stay dedicated to diurnal monitoring.

**Although they were score I or II, some patients were not subjected to clinical observation and received treatment.

Initial treatments consisted in corticosteroids for 39 patients (53.5%) and IVIG for 9 patients (12.3%) while a wait-and-watch approach was used in 25 patients (34.2%). Between 3 and 6 months of follow up, 2 patients received IVIG only, 4 received corticosteroids only and 2 received IVIG and corticosteroids. After 6 months, platelet count was <100.10^9^/L in 26/69 patients (37%) including 11/42 patients under 10 years (26.1%) and 15/27 patients over 10 years (55.5%) (p = 0.1).

### Health related quality of life

The main results concerning HRQL during the evolution of ITP are summarized in Table 3. The psycho-social summary score includes emotional, social and school functioning. Since we thought that change in scores between D1 and M6 were more suitable for assessing the evolution of HRQL, we mainly focused our analysis on these points.

**Table 3:** Health related quality of life scores for ITP children and adolescents

	**ITP diagnosis (n = 73)**						**ITP month 6 (n = 55)**				**Δ**	**Original norm data**^**1**^		**Healthy children, France**^**2 **^**(n = 185)**	
	**Mean**	**SD**	**% floor**	**% ceiling**	**Cronbach α coefficient**	**MCID**	**Mean**	**SD**	**% floor**	**% ceiling**		**Mean**	**SD**	**Mean**	**SD**
**Children**															
Global score	80.6	10.7	0.0	0.0	0.83	4.3	85.1	16.2	0.0	10.8	4.5*	83.8	12.6	77.6	12.5
Physical summary score	86.0	10.4	0.0	22.0	0.88	3.6	88.4	12.7	0.0	33.3	2.4	87.5	13.5	81.6	13.5
Psychosocial summary score	77.7	14.1	0.0	0.0	0.71	7.6	84.1	13.9	0.0	10.8	6.4	81.9	14.0	75.5	14.3
Emotional functioning	72.0	18.6	0.0	12.8	0.80	8.3	81.4	17.8	0.0	27.0	9.4*	79.3	18.1	69.8	18.4
Social functioning	86.4	16.2	0.0	30.0	0.81	7.0	90.7	13.8	0.0	48.6	4.3	85.1	16.7	80.5	17.9
School functioning	74.0	16.2	0.0	5.1	0.77	7.7	79.9	17.6	0.0	16.6	5.9	81.1	16.4	76.4	16.6
**Parent**															
Global score	82.7	12.3	0.0	4.1	0.85	4.6	87.2	12.5	0.0	14.5	4.5	82.7	15.4	80.4	12.1
Physical summary score	87.2	14.3	0.0	30.1	0.86	5.3	90.8	13.1	0.0	42.8	3.6	84.5	19.5	85.7	13.7
Psychosocial summary score	80.2	14.0	0.0	5.5	0.76	6.8	85.2	13.4	0.0	14.5	5	81.7	15.2	77.4	13.2
Emotional functioning	71.1	19.5	0.0	12.6	0.83	8.1	77.5	20.1	0.0	22.2	6.4	81.3	16.5	71.8	16.3
Social functioning	89.5	14.2	0.0	47.2	0.84	5.5	93.5	11.6	0.0	66.0	4	83.7	19.4	84.4	16.2
School functioning	80.6	18.1	0.0	24.6	0.82	7.6	86.1	14.3	0.0	30.1	5.5	78.8	19.6	76.1	16.7

% Floor/Ceiling = the proportion of scores at the extremes of the scaling range/SD = Standard deviation. Impaired health-related QOL was defined as a score that was more than 1 SD below the healthy sample mean/MCID = Minimal Clinically Important Difference. Standard Error of measurement computed by multiplying the standard deviation at diagnosis by the square root of 1 - alpha (Cronbach alpha coefficient)/Δ = the difference between mean scores at diagnosis and month 6. An improvement in quality of life between diagnosis and month 6 requires a Δ at least equal to the value of MCID score.

*p < 0.01.

^1^Varni et al., 2007: correspond to the standards obtained from a population of 5480 healthy US children ().

^2^Tessier et al., 2009.

We compared self-reported and parent-proxy global, physical and psycho-social mean scores of our ITP population to the mean scores of the US population (original norm data) and the mean scores of the French population. At study onset, no difference was found regardless of the healthy population reference since scores observed in the population on study did not range below SD compared to the healthy population scores. However, when compared to the US population, we noted lower mean scores at diagnosis in patients with ITP for emotional functioning and school functioning (respectively 72.6 versus 79.3 and 74 versus 81.1), but these results were not found when compared to the French population (respectively 72.6 versus 69.8 and 74 versus 76.4). In the same way, mean scores for parent-proxy reports, emotional functioning at diagnosis were lower than in the healthy population when compared to US population (71.1 versus 81.3) while they were the same when compared to the French population (71.1 versus 71.8). This reflects the fact that, in the French reference population, the mean values for emotional, social and school functioning are generally lower than that of the US population reference (except for social functioning in parent-proxy reports), maybe due to cultural differences between the two populations and to the fact that the two populations are not exactly similar in term of age.

We studied HRQL scores observed on D1 and M6. As previously defined, these changes were expressed in term of MCID, which measures more closely the impact of differences observed in quality of life. In children's reports, we observed a statistically significant improvement in HRQL scores from D1 to M6 only for global and emotional scores (Δ respectively 4.5 for an expected MCID score 4.3 and 9.4 for an expected MCID score 8.3). No statistically significant differences were found in parent-proxy reports (Δ < MCID for all items). The percentages of patients that presented a PedsQL score perceived to be beneficial (score at M6 at least equal to the value of MCID) in terms of global, physical and psychosocial score were respectively 54.5%, 35.6% and 36.2%. In the same way, the proportion of patients that presented a PedsQL score perceived to be beneficial in term of emotional, social and school functioning were respectively 42.1%, 28.5% and 32.1%.

There was no minimal floor effect for ITP children. However, ceiling effects existed. Ceiling effects for healthy children highly satisfied with their quality of life were reported in the literature but with much greater proportions compared to ITP children at diagnosis [21].

ICC between child and parent reports (Table 4) seemed better in M6 than at inclusion, with a better correlation in older children (more than 13 years old). For these teenagers, the main differences related to the physical score and the school functioning (upon inclusion) and the social and emotional functioning (in M6). The ICC between child self-report and parent proxy-report was measured as moderate to excellent.

**Table 4:** Agreement between child self-report and parent proxy-report

**Age**	**Global score ICC (95% IC)**	**Physical summary score ICC (95% IC)**	**Psycho-social summary score ICC (95% IC)**	**Emotional functioning ICC (95% IC)**	**Social functioning ICC (95% IC)**	**School functioning ICC (95% IC)**
**Inclusion**						
2 - 4 years	na	na	na	na	na	na
5 - 7 years	0.47 (0.11-0.86)	0.66 (0.28-0.92)	0.63 (0.27-0.90)	0.11 (0.01-0.90)	0.49 (0.12-0.86)	0.58 (0.21-0.88)
8 - 12 years	0.52 (0.23-0.80)	0.48 (0.18-0.80)	0.69 (0.47-0.87)	0.42 (0.14-0.76)	0.74 (0.52-0.89)	0.68 (0.43-0.86)
13 - 18 years	0.78 (0.53-0.93)	0.45 (0.11-0.84)	0.80 (0.56-0.94)	0.7 (0.52-0.93)	0.85 (0.65-0.95)	0.36 (0.32-0.88)
**Month 6**						
2 - 4 years	na	na	na	na	na	na
5 - 7 years	0.65 (0.23-0.93)	0.60 (0.18-0.92)	0.61 (0.24-0.89)	0.73 (0.42-0.92)	0.22 (0.07-0.93)	0.34 (0.10-0.90)
8 - 12 years	0.92 (0.82-0.97)	0.85 (0.69-0.94)	0.65 (0.39-0.85)	0.60 (0.32-0.83)	0.54 (0.25-0.80)	0.69 (0.40-0.89)
13 - 18 years	0.80 (0.56-0.94)	0.72 (0.42-0.91)	0.79 (0.53-0.94)	0.63 (0.29-0.88)	0.54 (0.13-0,89)	0.69 (0.36-0.91)

ICC (intraclass correlation coefficients) are designated as < =0.4 poor to fair agreement, 0.41-0.60 moderate agreement, 0.61-0.80 good agreement, and 0.81-1.00 excellent agreement.

To determine their influence on HRQL scores and MCID, several parameters were analyzed: gender, age at diagnosis, preceding viral infection, initial ICIS score, initial place of treatment, and platelet count on M6. Change in quality of life was measured by MCID score (Table 5). Patients with an initial hospitalization had a lower score in social functioning (75% vs 25%, p = 0.05) and patients with a preceding viral infection perceived an improvement of quality of life (73.3% vs 26.7%, p = 0.05) in term of global score, at least equal to the MCID score at M6. In contrast, there was no statistically significant difference related to gender, age and ICIS score at diagnosis or initial treatment. Platelet count at M6 (< or >100.109/L) did not affect the MCID either.

**Table 5:** Factors associated with Minimal Clinically Important Difference (MCID) between health related quality of life scores at diagnosis and scores at month 6

	**Global score**			**Physical summary score**			**Psychosocial summary score**			**Emotional functioning**			**Social functioning**			**School functioning**		
	**β**	**SE**	**p**	**β**	**SE**	**p**	**β**	**SE**	**p**	**β**	**SE**	**p**	**β**	**SE**	**p**	**β**	**SE**	**p**
**Gender**																		
Female	0.32	0.54	0.55	0.28	0.54	0.59	−0.06	0.54	0.90	−0.01	0.53	0.97	−0.02	0.59	0.73	−0.54	0.57	0.34
Male	Ref			Ref			Ref			Ref			Ref			Ref		
**Age**																		
2 - 4 years	0.51	0.63	0.41	0.44	0.59	0.45	1.05	0.71	0.14	0.65	0.59	0.27	−0.24	0.60	0.68	0.92	0.68	0.17
5 - 7 years	−0.02	0.72	0.96	1.28	1.08	0.23	0.74	0.97	0.45	1.28	1.08	0.23	1.08	1.03	0.29	−0.03	1.02	0.97
8 - 12 years	−0.35	0.58	0.54	0.13	0.41	0.75	−0.47	0.45	0.29	0.10	0.40	0.80	−0.34	0.44	0.44	−0.05	0.46	0.99
13 - 18 years	−0.69	0.57	0.22	−0.24	0.49	0.61	−0.44	0.51	0.38	0.66	0.58	0.25	−0.37	0.54	0.49	−1.52	0.83	0.06
Age < =10 years	0.77	0.57	0.18	−0.22	0.54	0.68	−0.28	0.55	0.60	−0.55	0.54	0.30	0.20	0.60	0.73	0.01	0.57	0.98
**Preceding viral infection**	1.25	0.57	0.03	0.48	0.58	0.4	0.78	0.61	0.20	−0.18	0.56	0.74	−0.96	0.61	0.11	0.82	0.65	0.20
**ICIS scores**																		
Score I	Ref			Ref			Ref			Ref			Ref			Ref		
Score II	−15.2	109	0.98	14.5	198	0.99	14.4	208	0.99	14.8	203	0.99	15.4	331	0.99	13.52	167	0.99
Score III or IV	−14.9	109	0.98	14.6	198	0.99	14.9	208	0.99	15.01	203	0.99	15.25	331	0.99	14.39	167	0.99
**Initial place of treatment**																		
Inpatient	0.15	0.87	0.86	−0.87	0.74	0.23	−0.39	0.74	0.59	0.53	0.77	0.48	−1.45	0.77	0.05	1.49	1.11	0.17
Observation unit	0.22	1.05	0.83	−1.32	1.05	0.21	−0.87	1.05	0.40	0.18	1.01	0.85	−1.83	1.28	0.15	1.16	1.35	0.38
Outpatient	Ref			Ref			Ref			Ref			Ref			Ref		
**Initial treatment**																		
Corticosteroid	0.81	0.60	0.17	−0.02	0,59	0.96	0.77	0.63	0.22	0.13	0.58	0.81	−0.02	0.66	0.96	1.21	0.72	0.09
IVIG	0.60	0.89	0.49	0.33	0.89	0.71	0.81	0.92	0.37	0.11	0.88	0.89	1.02	0.96	0.28	1.67	1.03	0.10
No treatment	Ref			Ref			Ref			Ref			Ref			Ref		
**Platelets**																		
Platelets >100000 at month 6	0.67	0.57	0.23	0.12	0.56	0.82	0.20	0.57	0.71	−0.19	0.56	0.72	0.21	0.62	0.73	0.65	0.59	0.27

ref = reference/β coefficients and SEs from logistic regression such that positive β coefficient means that predictor or increasing values of predictor are associated with better quality of life with MCID.

## Discussion

In recent years, measurement of HRQL has become more important in the assessment of several pediatric diseases, including ITP. The lack of evidence that any of the current treatments can modify either the short or long-term outcomes in children with ITP generated interest in assessing HRQL in this population. This study aimed to determine the impact of ITP on children’s HRQL from diagnosis to six months after diagnosis and to identify any risk factors.

Considering the first objective, we observed a slightly lower score for baseline emotional functioning in parent-proxy and self reports as well as the school functioning in self-reports when compared to original norm data. Surprisingly, this difference was not found when the comparison was performed with the French healthy population [22]: results were generally even higher than in the healthy population for most items. The fact that children enrolled in the French reference population were older than those of our study could be one of the possible explanation for this difference, as younger children are less likely to worry. But this does not explain all the differences observed in parent-proxy reports. These differences were not sufficient to be considered as an impairment, nor a minimal clinically important in terms of quality of life. We did not observe any difference of HRQL between self or parent-proxy-reports and the original norm data after 6 months of ITP evolution either. The results are not surprising as ITP is a blood disease which causes considerable worry about both rare but serious bleeding and having a more serious condition. Over the following months, most patients (children and parents) experienced an improvement of HRQL and at M6 quality of life was the same as the healthy population. On all major parameters, HRQL scores increased between D1 and M6 and, considering the global score, the majority of patients (54.5%) perceived a change in quality of life as measured by MCID. This was an expected result as it coincides with the natural favorable evolution of the disease [8-27]. In our cohort, as in previous reports, about two thirds of children had recovered a normal platelet count at 6 months and bleeding scores improved with time in the majority of the patients. We can also hypothesize that the knowledge concerning ITP that parents and children develop over time, helps reduce their anxiety [28]. Interestingly, significantly lower ICC was noted for children of 5 to 7 years old, especially for emotional functioning at diagnosis and for social and school functioning at M6, as already mentioned in the literature [22]. More generally this difference is observed in the global score with better agreement for children of more than 13 years old. Although MCID does not significantly differ depending on age, these data could suggest a trend for young children to be more affected by ITP. The strict limitation of the child’s activities enforced by the parents to protect them from serious injuries could be one of the major reasons for their stress and their parents’ concern, which lead to a lower score. On the other hand, in older children, one can also speculate that HRQL self-report results are a representation of the sense that teenagers might believe themselves to be invincible.

Considering the second end-point, from our results, a lower score in social functioning seemed to be associated with an initial hospitalisation and the quality of life perceived in term of global score at M6 was associated with a viral infection before ITP (Table 5). Other variables such as age, platelet count at diagnosis and 6 months, initial bleeding scores or initial treatment did not influence scores of children or parents. This result is consistent with the findings of Neunert et al. who found only weak and non-significant negative correlations between bleeding severity or platelet count at diagnosis and HRQL [14]. In our series about two thirds of patients had an initial ICIS score ≥ III with a platelet count < 10.10^9^/L, with both variables improving with time, lowering anxiety of parents and children. Conversely, a recent viral infection could potentially have symptoms affecting the child’s HRQL, with a subsequent improvement in HRQL once the infection resolves, explaining the significant change in the MCID between D1 and M6. Similarly, the initial hospitalization would impact on HRQL, regardless of the reason for hospitalization. So, these two factors (viral infection and initial hospitalization) appear to effect the MCID independently of ITP.

Some limitations in our study should be pointed out. First, the limited size of our cohort did not allow us to analyse clinically relevant subgroups of patients such as those with persistent ITP at M6, with regular physical activity or patients from different socio-professional origins. Second, patients were recruited from seven pediatric treatment centers. These various origins may have induced a clustering effect of HRQL according to the medical and paramedical speech at diagnosis and through-out the follow-up. Third, we did not differentiate assessments of mothers and fathers. We can speculate that parents can have different evaluations of their own child quality of life. Fourth, a significant number of patients had an initial ceiling effect at 100: in such a condition, we cannot detect any improvement in quality of life during follow-up. Finally, although PedSQL™ 4.0 is an international tool, translated and linguistically validated in French, there is, to our knowledge, only one study establishing references in a French healthy population of 185 children [22]. We compared our results to those published in the larger series published by Varni et al. [11]: differences observed (Table 3) raise the question of the validity of some of our comparisons especially as the validation performed by Tessier et al. [22] was conducted among school children (over 5 years). As emphasized by these authors, further work would probably be required in the French population to determine real reference values for PedSQL™ 4.0.

However, despite these limitations, we think it important to note that ITP does not significantly affect HRQL as measured by the PedSQL™ 4.0. Even if we observe an initial decrease in scores (especially in the psychosocial field), it remains moderate and quickly corrected within 6 months after diagnosis. We can wonder if the PedsQL™ 4.0 Generic Core Scales is a sufficient tool to measure HRQL in children with ITP. Recently, Klaassen et al. [16] performed a cross-culturally translated KIT (Kids’ ITP Tools) validation. This tool is specifically designed to measure HRQL in children with ITP. Its validity was assessed by a recent study in four countries and French newly diagnosed patients, different from those reported here, were enrolled in this study [16]. In the whole study population, the authors note that the KIT scores are moderately correlated with the PedsQL scores. Taking into account these results and our own results, we think it would be interesting to study the quality of life of these patients with a larger cohort.

## Conclusions

This longitudinal study assessing HRQL in children with ITP reveals a global improvement in PedSQL™ 4.0 scores from diagnosis up to six months of follow up, but these variations are not sufficient to indicate significant changes in quality of life. Our data also confirm that self-reported HRQL scores are not influenced by any analyzed biological or clinical parameters. However, further studies using a disease-specific measure, such as the Kids’ ITP Tools, will certainly provide more information on HRQL.

## Abbreviations

HRQL: Health related quality of life; ICC: Intraclass correlation coefficient; ICIS: Intercontinental childhood ITP study group; ITP: Immune thrombocytopenia; IVIG: Intravenous immunoglobulin; KIT: Kids’ ITP tools; MCID: Minimal clinically important difference; SD: Standard deviation; SEM: Standard error of measurement.

## Competing of interests

The authors declare no competing financial interests.

## Authors’ contributions

MS wrote the manuscript, carried out the initial analyses, and approved the final manuscript as submitted. IP conceptualized, designed and supervised the study, designed the data collection instruments, designed the data collection instruments, carried out the initial analyses, drafted the initial manuscript, and approved the final manuscript as submitted. JR carried out the initial analyses, drafted the initial manuscript, and approved the final manuscript as submitted. ET conceptualized, designed the study, coordinated and supervised data collection, drafted the initial manuscript, and approved the final manuscript as submitted. CS carried out the initial analyses, and approved the final manuscript as submitted. CT conceptualized, designed the data collection instruments, coordinated and supervised data collection, and approved the final manuscript as submitted. FM, YR, SP, AB, JB, DM, PR coordinated and supervised data collection, critically reviewed the manuscript, and approved the final manuscript as submitted. EQ designed the data collection instruments, critically reviewed and approved the final manuscript as submitted. NLY and RJK: designed the data collection instruments, critically reviewed and revised the manuscript, and approved the final manuscript as submitted.
